# Oropharyngeal Syphilis Presenting as Tongue Base Ulcer With Lymphadenopathy

**DOI:** 10.1155/crot/2673365

**Published:** 2026-05-19

**Authors:** Serena F. Pu, Kevin J. Carlson, Jonathan R. Mark, Benjamin J. Rubinstein

**Affiliations:** ^1^ Department of Otolaryngology–Head and Neck Surgery, Macon and Joan Brock Virginia Health Sciences EVMS Medical Group at Old Dominion University, Norfolk, Virginia, USA

**Keywords:** lymphadenopathy, syphilis, tongue base ulcer

## Abstract

Within the aerodigestive tract, syphilis presents with varied signs and symptoms, some of which may mimic malignancy or other disease processes. We present the case of a 29‐year‐old man with otalgia and odynophagia who was found on physical examination and office laryngoscopy to have a 4‐cm ulcerative mass of the tongue base and cervical lymphadenopathy. While planning for tissue diagnosis, the patient’s serologies revealed positive treponemal antibody testing and elevated rapid plasma reagin (RPR) titers, confirming syphilitic infection, likely representing secondary syphilis. While awaiting operative biopsy, the patient received intramuscular penicillin with complete resolution of the mass and symptoms, and ultimately operative intervention was deferred. While planning for biopsy of suspicious head and neck lesions, clinicians should consider infectious disease workups in patients with risk factors.

## 1. Introduction

Syphilis, caused by the spirochete *Treponema pallidum*, is often referred to as the “great imitator” due to its varied presentation [1, 2]. The incidence of syphilis in the United States continues to rise in sexually active patients, with the Centers for Disease Control and Prevention citing over 171,000 cases in 2021 from just over 101,000 cases in 2017 [3]. Within the aerodigestive tract, chancres, gummas, and ulcers can present with a variety of symptoms depending on the anatomical sites involved [1]. These lesions can be easily confused for alternate pathologies in the head and neck, such as malignancy [4, 5]. We report a case of syphilis presenting as an ulcerative mass of the tongue base and associated cervical lymphadenopathy.

## 2. Case Presentation

**FIGURE 1:**
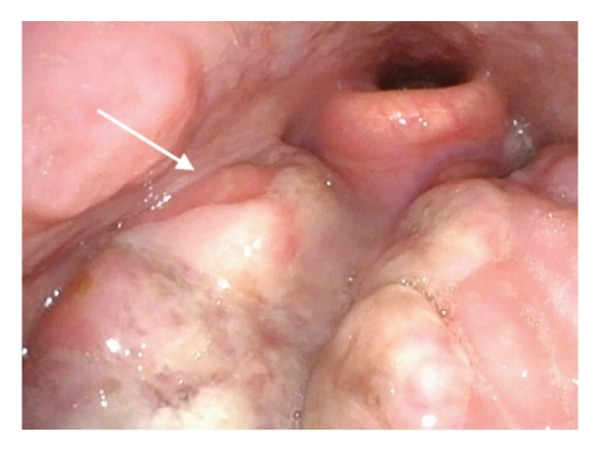
Lesion at right tongue base, seen on initial laryngoscopy.

A 29‐year‐old male was referred by his primary care physician to an otolaryngology clinic for 3 months of otalgia and odynophagia. He described waxing and waning right‐sided cervical lymphadenopathy and one episode of hemoptysis but denied recent or ongoing fevers, chills, night sweats, diffuse rash, or unexplained weight loss. His past medical history was notable for recently treated oropharyngeal chlamydia. His social history was significant for a 5‐pack‐year smoking history, daily marijuana use, and social alcohol use. On physical examination, he had multiple firm, nontender, enlarged right‐sided cervical lymph nodes (greatest ∼3 cm) and a firm mass of the right tongue base. Flexible laryngoscopy revealed an ulcerative mass measuring 4 cm at the right tongue base (Figure 1). Computed tomography (CT) imaging with contrast of the neck showed multiple enlarged bilateral Level II lymph nodes, measuring up to 22 mm on the right and 16 mm on the left, without central necrosis. There were no other significant findings in the pharynx or larynx.

**FIGURE 2:**
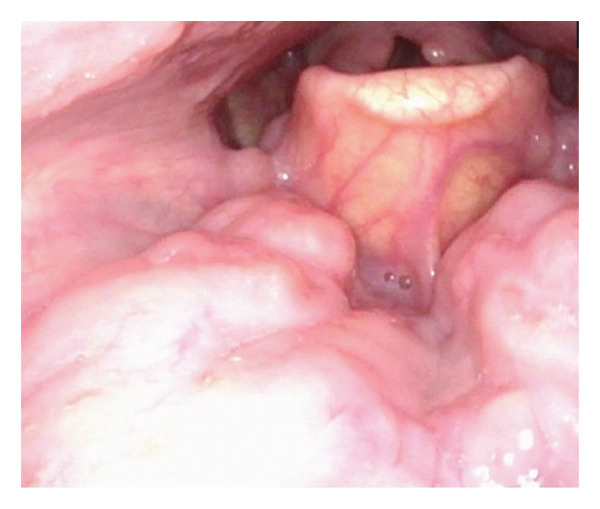
Right tongue base after treatment with intramuscular benzathine penicillin.

The remaining head and neck examination was unremarkable. While planning for biopsy, serologies for infectious diseases were ordered. Treponemal antibody testing was positive, and the rapid plasma reagin (RPR) titer was 1:64, confirming a diagnosis of syphilis. HIV testing was negative. The patient began empiric treatment with intramuscular penicillin G per World Health Organization (WHO) guidelines [6]. His symptoms and cervical lymphadenopathy resolved within 2 weeks, and the patient elected to cancel his scheduled biopsy in favor of close follow‐up. Three weeks after treatment, RPR titers decreased appropriately 32‐fold to 1:2, and repeat in‐office examination and laryngoscopy demonstrated stable resolution of the lesion (Figure 2). Biopsies were not pursued due to complete resolution of the tongue base lesion and lymphadenopathy. His titers were followed for the subsequent 24 months and remained low at 1:1 throughout follow‐up, with no recurrence of symptoms or lymphadenopathy. Patient consent was obtained for the presentation of this case.

## 3. Discussion

This case, which was suspicious for tongue base malignancy, demonstrates one way syphilis can present within the head and neck. Syphilis presents in three classic stages. Primary syphilitic lesions are typically transient ulcers (chancres) involving the genitalia that resolve within weeks, though they can occur in the oral cavity or pharynx. Secondary syphilis results from systemic dissemination of disease, which may include a disseminated maculopapular rash involving the palms and soles, fever, malaise, condyloma lata, and diffuse lymphadenopathy. Tertiary syphilis involves the cardiovascular system or central nervous system and can cause granulomatous lesions of the skin and soft tissues (gummas) [6]. Other important differential diagnoses include discoid lupus, sarcoidosis, atypical mycobacterial infections, lymphomas, and other malignancies [7]. Due to the transience of primary lesions, many patients are diagnosed at the secondary stage [5]. Although it is possible that the above patient’s lesion represented primary syphilis with an atypical presentation, the high RPR titers (typically 1:32 or greater), a lack of a primary chancre, and lymphoid manifestations are more consistent with the secondary stage [8]. The tongue base ulcer may represent secondary lymphoid involvement or condyloma lata, and his lymphadenopathy is a sign of disease dissemination.

Diagnosis is primarily established via serology using two main algorithms: standard screening, in which a reactive nontreponemal test is followed by treponemal testing to confirm; or reverse‐screening, in which a reactive treponemal test is followed by nontreponemal testing to obtain titers. Nontreponemal tests include RPR and venereal disease research laboratory (VDRL) tests, and treponemal tests include fluorescent treponemal antibody absorption (FTA‐ABS) and treponemal IgG/IgM. Treponemal antibodies are best used for symptoms of primary and early secondary syphilis, whereas nontreponemal serologies peak as secondary syphilis symptoms wane [9]. The patient above had titers drawn in a combination approach, where his positive treponemal antibody testing was automatically reflexed to assess nontreponemal RPR titers. Visualization of spirochetes under dark‐field microscopy is also diagnostic but has low utility in oral lesions [9]. Imaging and tissue sampling may be necessary in atypical cases, but serologic screening represents the fastest and least invasive first step [10]. After diagnosis, standard treatment is penicillin G, and symptoms typically resolve within weeks. A four‐fold change in titers is the standard for demonstrating treatment response [11]. The patient above had prompt resolution of his lesion and appropriate decrease in his titers, he and elected for close follow‐up rather than tissue sampling.

Aerodigestive lesions with associated lymphadenopathy may be a presentation of squamous cell carcinomas (SCCs) of the head and neck, and previous cases reinforce the similar presentation of oropharyngeal SCC and syphilitic lesions [2, 4, 5, 12]. According to a 2024 review by Guarino et al., in the head and neck, the oropharynx is an uncommon site of infection of syphilis overall and in the head and neck (the oral cavity represents the most common site) [7]. However, infectious etiologies including syphilis, HIV, EBV, HSV, oral candidiasis, and tuberculosis should be entertained when risk factors are present. In syphilis specifically, HIV coinfection, tobacco use, drug misuse, and men who have sex with men (MSM) are risk factors [7]. The patient in the presented case did not have a clearly documented sexual history but did have a history of oropharyngeal chlamydia which had been treated. While the American Academy of Otolaryngology‐Head and Neck Surgery clinical practice guideline recommends that practitioners consider infectious etiologies in the evaluation of neck masses in an adult, syphilis is absent from the list of commonly ordered bacterial or viral titers [13]. Both syphilis and HPV‐associated oropharyngeal SCC are increasing in incidence nationally and share sexual activity as a common risk factor [14, 15]. Therefore, lesions of the aerodigestive tract and/or cervical lymphadenopathy should raise suspicion for both conditions in sexually active patients, and prompt identification and treatment of syphilis can avoid unnecessary operative intervention.

## Funding

No funding was received for this manuscript.

## Conflicts of Interest

The authors declare no conflicts of interest.

## Data Availability

The data that support the findings of this study are available on request from the corresponding author. The data are not publicly available due to privacy or ethical restrictions.
